# Prediction and Experimental Verification of a Hierarchical Transcription Factor Regulatory Network of Porcine Myoglobin (Mb)

**DOI:** 10.3390/ani11123599

**Published:** 2021-12-19

**Authors:** Di Yuan, Hao Yu, Songcai Liu, Linlin Hao, Jing Zhang

**Affiliations:** College of Animal Science, Jilin University, 5333 Xi’an Road, Changchun 130062, China; yuandi19@mails.jlu.edu.cn (D.Y.); yu_hao@jlu.edu.cn (H.Y.); songcai@jlu.edu.cn (S.L.)

**Keywords:** meat color, myoglobin, Bama pigs, Yorkshire pigs, transcription factor

## Abstract

**Simple Summary:**

Myoglobin (*Mb*) is the sarcoplasmic heme protein primarily responsible for meat’s color. The transcription pattern of the porcine *Mb* gene has not been studied because its genome structure information has not been officially annotated. In our study, we attempted to reveal the possible mechanism of pig-meat color formation by integrating the public data of genome and transcriptome.

**Abstract:**

Myoglobin is a key chemical component that determines meat’s color and affects consumers’ purchase intentions. In this work, we firstly identified the promoter sequence of the *Mb* gene from the primary assembly of high-throughput genome sequencing in pigs, and predicted its potential transcription factors by LASAGNA. Through the data mining of the mRNA expression profile of longissimus dorsi muscle of different pig breeds, we constructed a hierarchical interplay network of Mb-TFs (Myoglobin-Transcription Factors), consisting of 16 adaptive transcription factors and 23 secondary transcription factors. The verification of gene expression in longissimus dorsi muscle showed that the *Mb* mRNA and encoded protein were significantly (*p* < 0.05) more abundant in Bama pigs than Yorkshire pigs. The qRT-PCR (Real-Time Quantitative Reverse Transcription PCR) validation on genes of the Mb-TFs network showed that *FOS*, *STAT3*, *STAT1*, *NEFL21*, *NFE2L2* and *MAFB* were significant positive regulatory core transcription factors of Mb-TFs network in Bama pigs, whereas *ATF3* was the secondary transcription factor most responsible for the activation of the above transcription factors. Our study provides a new strategy to unravel the mechanism of pork color formation, based on public transcriptome and genome data analysis.

## 1. Introduction

With the development of society and the improvement of human living standards, people’s focus on meat consumption has gradually shifted from “quantity” to “quality” [1]. The Western commercial crossbred pig Duroc × Landrace × Yorkshire (DLY) has the advantages of high feed conversion efficiency and a fast growth rate, and the consequent economic benefits, in addition to sharing the largest market in China and the world [2]. DLY meat is frequently classified as low quality by consumers, which is explained by the pig’s rapid growth, which results in PSE (pale, soft and exudation) or DFD (black, hard and dry) meat. However, some native pig breeds have become popular in the market because of their good meat quality [3,4]. Meat quality is a broad term that can be defined by several different attributes, such as pH, drip loss and color. Among these, meat color is one of the most important factor that affects the purchase decision [5], since consumers are unable to evaluate the taste or feel the texture of meat without opening the package [6]. According to research statistics, the price discount caused by meat color brings serious economic losses and a sustainable negative impact on the environment to the meat industry every year [7,8].

The different degrees of meat color depend on *Mb* content and chemical form [9]. *Mb* in meat exists in three forms: oxygenated oxymyoglobin (*OxyMb*), oxidized metmyoglobin (*MetMb*) and reduced deoxymyoglobin (*DeoMb*) [10]. Different forms of *Mb* constitute different meat colors valued by the consumer when buying meat. Consumers generally prefer the bright red flesh color formed by muscle-oxygenated protein [11]. The global demand for meat continues to rise, and is projected to reach 90 kg per capita by 2050 [12]. Pork is preferred by the majority of meat consumers, and accounted for 42.2% (110.6 million metric tonnes) of global meat consumption in 2017 [13]. In light of the challenges in meeting the increasing global demand, waste within the distribution and consumption phases of the food supply chain is of particular concern. More than half of edible meat products are wasted, and meat discarded at these two phases of the supply chain are done so to satisfy consumer perception of meat quality [14]. The lipid oxidation of meat products is primarily responsible for off-flavors and undesirable changes in color that characterize the reduction in quality and storage stability [15]. Several researchers have demonstrated that myoglobin oxidation and lipid oxidation are interrelated, and that the acceleration of one will exacerbate the other [16,17]. Studies have shown that the oxidation of unsaturated fatty acids can mediate the conversion of oxygenated oxymyoglobin to metmyoglobin and make the meat grayish brown. This usually becomes the subjective basis for consumers to judge it as inferior meat [18].

*Mb* is an ubiquitous protein found in striated muscle of nearly all vertebrate taxa. Studies have shown that the heritability estimate for porcine longissimus myoglobin concentration was 0.27, with moderate heritability [19]. The observation of the differential expression pattern of *Mb* during myogenesis and in various adult striated muscle-fiber types has prompted investigators to study the putative myoglobin promoters and the various transcription factors that are responsible for the regulation of *Mb* transcription. Using cultured adult myoblasts obtained from 11 to 12-day-old chicken embryonic pectoral muscle, researchers have demonstrated that a 167 bp myoglobin promoter region (−371 to −205), containing a 57 bp cis-acting element, enhanced reporter activity in differentiated myotubes and concluded that the 2 kb putative *Mb* promoter region contains a muscle-specific enhancer region that is essential for the regulation of *Mb* transcription during skeletal muscle cell differentiation [20]. So far, porcine *Mb* gene has not been identified in the reference genome, and its transcriptional regulation pattern has not been studied, due to the lack of sequence information of the promoter region [20,21].

In order to investigate the *Mb* genome location and its transcription pattern in pigs, we performed a data mining of pig genomic and transcriptomic data available in a public database, in addition to revealing the influence of the *Mb* transcription regulation pattern in pork color formation through in vitro validation.

## 2. Materials and Methods

### 2.1. Animal Collection and RNA Extraction

Three male Bama pigs (~45 kg for 6 months old) and three male Yorkshire pigs (~100 kg for 6 months old) were selected separately for slaughter; tissue samples from the sixth to seventh back ribs of the longissimus dorsi muscle, and the total RNA, were extracted and quantified. The Reverse Transcription Kit (PrimeScript™ RT reagent Kit with gDNA Eraser, Takara, Kusatsu, Japan) was used to remove genomic DNA at 42 ℃ for 2 min. The samples were directly retrotranscribed for 15 min to convert total RNA to cDNA, for the post-study of *Mb* and its related transcription factors.

### 2.2. In Silico Analysis of the Porcine Mb Promoter to Identify Transcription Factors Binding Sites

The genomic information of the *Mb* gene, including all exons and introns, can be retrieved from the primary assembly of Wuzhishan minipigs in the Ensembl database (http://asia.ensembl.org/index.html (accessed on 15 May 2021)). A region ~2 kb upstream of the transcription start site of the *Mb* gene is defined as a potential promoter for the prediction of transcription factor-binding sites. The putative transcription factor binding sites (TFBSs) in this region were analyzed using the online analysis server LASAGNA (https://biogrid-lasagna.engr.uconn.edu/lasagna_search/ (accessed on 15 May 2021)). We used JASPAR CORE matrices as matrix-derived models for transcription factors, and the filter for the predicted results was the cut-off value: *p* < 0.01.

### 2.3. Identification of Differentially Expressed Genes (DEGs)

The gene expression profiling datasets GSE119368 and GSE99092 were downloaded from the Gene Expression Omnibus (GEO) database. DEGs between local and commercial pig breeds were analyzed with the DESeq2 package in a Bioconductor (version 3.8) [22], with a cut-off threshold of *p* < 0.05 and a fold change ≥2.0.

### 2.4. Functional Enrichment Analysis of DEGs

To elucidate potential biological processes, Yu et al. conducted the Kyoto Encyclopedia of Genes and Genomes (KEGG) pathway enrichment analysis, which was carried out by clusterProfiler to expound the promising signaling pathways correlated with the core transcription factors and secondary transcription factors [23]. The adjusted *p*-value < 0.05 was defined as the cut-off criteria.

### 2.5. Mb-TFs Interaction Network (TTI) Construction

The core TF (Transcription Factor)-secondary TF interaction network was constructed using the STRING online database. TTI (Transmission Time Interval) pairs with a combined score ≥ 0.4 were used to construct the TTI network. Transcription factors were annotated by TRRUST v2 [24]. Then, the regulatory relationship between TFs was visualized using Cytoscape software (version 3.8.2) (Institute of Systems Biology, Seattle, DC, USA).

### 2.6. Meat Color Apparent Index Determination

The value of meat color was measured by the Germany Matthaus meat color tester Opto-Star, and the Opto value (colorimetric value) was used to evaluate the color score of pork. The “OPTO-STAR” gun is an intelligent probe for measuring the brightness of meat, whose multiple functions greatly assist the user’s task. As the measurement method, the measurement head was placed on the cross section of the longissimus dorsi muscle and the measurement was completed in 3 s. In the “MEASURE” routine, we used the calibration-bloc for simulating a meat-measurement. We put the sensor onto color “0”, and left it for three seconds. The display then counts down to zero: if it is near to zero (+1/−1) then the bloc is recognized from the system; press “ENT”. We repeated the procedure with the second color “90”. After this, the system checked plausibility and jumped automatically back to “MEASURE”, if calibration was done properly. The measurement was carried out 24 h after slaughter. Opto value reference standard: Excellent: Opto-wert ≥ 63; Good: 53 ≤ Opto-wert < 63; Inferior: Opto-wert ≤ 53.

### 2.7. Western Blotting

Western blotting was performed on the same set of samples used in the DIGE (Dynamic Interactive Gerenal Equilibrium) analysis. Briefly, 50 μg pooled protein extracts of each group were fractionated by 10% SDS-PAGE (Sodium salt-Polyacrylamide gel electrophoresis) and transferred to a PVDF (Polyvinylidene Fluoride) membrane as previously described [25]. The signal was detected with an enhanced chemiluminescence (ECL) plus kit (Milipore, Bedford, MA, USA). Anti-manganese superoxide dismutase (anti-MnSOD) (1:2000), anti-heat shock protein B1 (anti-HspB1) (1:1000), anti-fatty acid binding protein 5 (anti-FABP5) (1:1000), and anti-glycerol-3-phosphate dehydrogenase (anti-GAPDH) (1:1000) were used. All of these antibodies were rabbit polyclonal antibodies, and were purchased from Abcam, Inc. (Cambridge, MA, USA).

### 2.8. Quantitative Real-Time PCR

The quantity and purity of the samples were assessed with a Nanodrop ND-2000 spectrophotometer (Thermo Scientific, Waltham, MA, USA). The qRT-PCR reaction was performed on an ABI PRISM 7900HT thermal cycler (Applied Biosystems, Waltham, MA, USA) using FastStart Universal SYBR Green Master (Thermo Scientific, Waltham, MA, USA). Each sample was repeated three times, and the data represent the average of at least three measurements. The Ct value of the target gene was normalized with the average RNA expression of the housekeeping gene *β-actin* [26,27]. The primers were designed using the primer3 web server (https://primer3.ut.ee/ (accessed on 12 March 2021) and shown in Table S1.

### 2.9. Statistical Analysis

Statistical analyses were performed using the software GraphPad Prism for Windows (GraphPad Software, version 8.0.1, GraphPad Software, San Diego, CA, USA). Values were expressed as the mean ± SD. Comparisons between two groups were performed by Student’s *t*-tests. A value of *p* < 0.05 indicated a significant difference.

## 3. Results

### 3.1. The Localization of the Mb Gene on the Porcine Reference Genome

**Figure 1:**
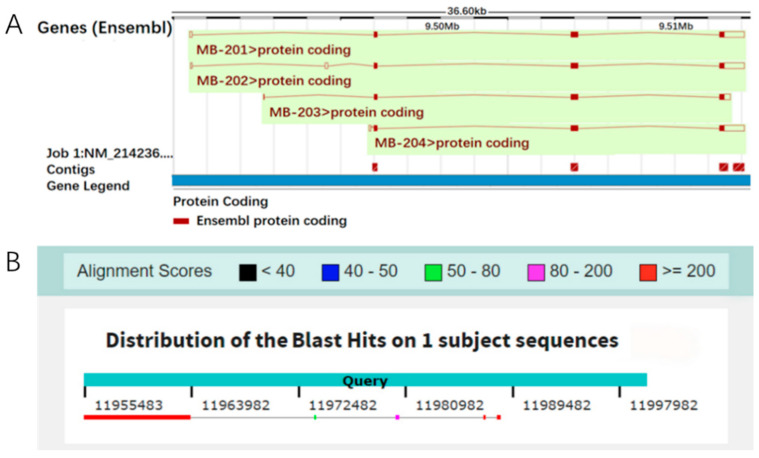
The localization of *Mb* gene on the porcine reference genome. (**A**) genomic structure information of *Mb* transcripts on the primary assembly of Wuzhishan mini-pig; (**B**) the localization of *Mb* gene on chromosome 5 of the porcine reference genome.

**Figure 2:**
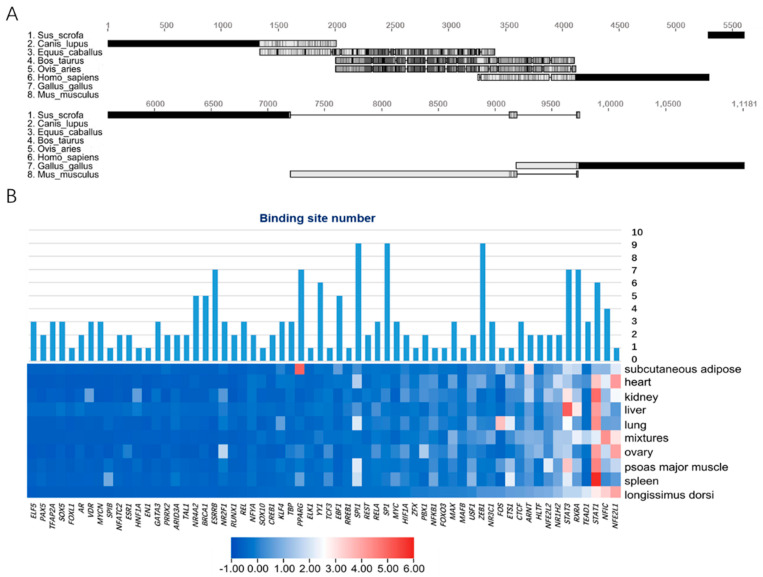
Prediction and enrichment of transcription factors of porcine *Mb* gene. (**A**) multiple sequence alignment of promoters (2 kb) of *Mb* gene in 8 representative mammals; (**B**) heatmap of 62 TFs expression in 9 tissues of pig with binding sites integrated.

Currently, the *Mb* gene has no annotation information on the official porcine reference genome. We performed the homology alignment of the *Mb* mRNA sequence on the primary assemblies of 12 pig breeds, and found that the *Mb* gene had complete genomic information on the primary assembly (primary assembly KQ001810.1:9,495,496–9,512,093) of the Wuzhishan mini-pig (Figure 1), while its transcriptional start on the reference genome corresponded with the loci of 11,955,483 on chromosome 5, but only extended 8528 bp. The other 8113 bp may have been lost due to the mis-assembly of the genome, as shown in Figure 2.

### 3.2. Prediction and Enrichment of Transcription Factors of Porcine Mb Gene

We selected the upstream 2 kb sequences of eight representative mammalian *Mb* genes as promoter regions for multiple sequence alignment. The results showed that the promoter regions of cattle and sheep were highly similar and shared ~1.5 kb homologous sequences with horses. However, pigs did not have highly similar fragments with other species, which indicated that the transcription pattern of *Mb* genes of pigs was independently evolved by specific selection (Figure 2A).

Secondly, we used a LASAGNA-Search 2.0 web server to identify 98 potential vertebrate transcription factors in the promoter region of porcine *Mb* gene, of which 86 could find orthologous genes in pig genomes (Table S1). The expression profile of pig tissue (GSE123858) showed in the longissimus dorsi tissue, but 24 TFs were not expressed at all. We constructed an expression profile heatmap of 62 TFs, and integrated their binding numbers in the promoter region of the *Mb* gene. The integrated heatmap showed that *STAT3*, *RXRA* and *STAT1* were not only highly expressed, but also had more binding sites than most other TFs.

### 3.3. Prediction of Adaptive Transcription Factors of Porcine Mb Gene and Construction of Its Interaction Network

**Figure 3:**
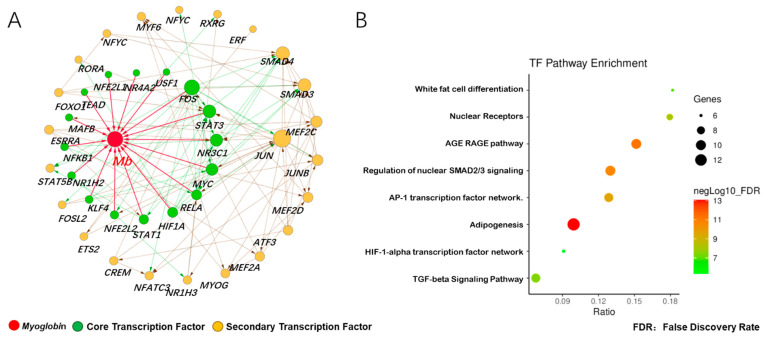
Prediction of adaptive transcription factors of porcine *Mb* gene and construction of its interaction network. (**A**) transcription factor interaction network of the porcine *Mb* gene. The network is constructed using the interaction data from the STRING database via the Cytoscape software. Red, myoglobin; green, core transcription factor; orange, secondary transcription factor. The size of the node (protein) indicates node connection degree (interaction relationships); (**B**) pathway enrichment of transcription factors of the porcine *Mb* gene.

According to Figure 2B, we found that the expression of 62 transcription factors in various tissues had a similar trend; we therefore speculated that these transcription factors played a role by constitutive expression or adaptive expression. In order to explore the potential transcription factors for the adaptive expression of the *Mb* gene, we first selected two data sets (GSE119368:DPL vs. Landrace, GSE99092:Wei vs. Yorkshire) from the GEO database, and analyzed the gene differential expression. The results showed that 2620 up-regulated DGEs in muscle tissue of Dapulian pigs included 74 transcription factors, while 493 up-regulated DGEs in muscle tissue of Wei pigs included 13 transcription factors. Among the 87 activated transcription factors, 16 core transcription factors were found to bind to the promoter region of *Mb* gene, and 23 secondary transcription factors interacted with the *Mb* gene and its core transcription factors (Figure 3A). The analysis of the KEGG pathway enrichment further showed that the above transcription factors mainly participated in eight signal pathways, with the highest enrichment ratio being white adipocyte differentiation, and the highest enrichment gene number being the adipogenesis pathway (Figure 3B). These results indicated that transcription factors in skeletal muscle (longissimus dorsi) of Chinese local pig breeds can not only activate *Mb* gene, but also promote the lipid metabolism of skeletal muscle cells.

### 3.4. Detection of Meat Color and Mb Gene Expression

**Figure 4:**
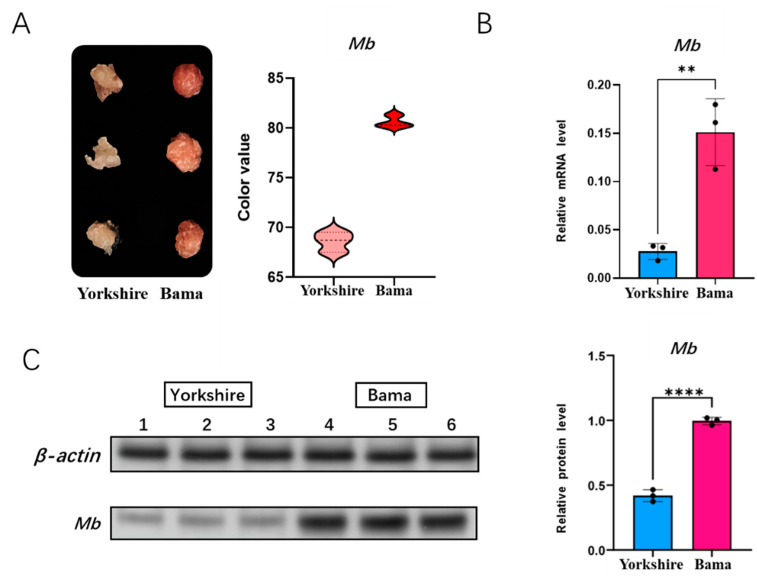
Detection of meat color and *Mb* gene expression. (**A**) meat color detection of Yorkshire and Bama as measured by the Germany Matthaus meat color tester Opto-Star; (**B**) *Mb* mRNA expression of Yorkshire and Bama as measured by RT-qPCR; (**C**) *Mb* protein expression of Yorkshire and Bama as measured by Western blot (Original Western Blot Figures are in Figure S1). ** *p* < 0.05, **** *p* < 0.01.

We first measured the meat color of Bama pigs and Yorkshire pigs by the Germany Matthaus meat color tester Opto-Star. The meat color tester assessment showed that Bama pigs had significantly higher specific color values than Yorkshire pigs, indicating that their *Mb* content was higher than that of Yorkshire pigs (Figure 4A). In order to identify the mRNA expression level of the *Mb* gene between Bama pigs and Yorkshire pigs, we performed qRT-PCR in longissimus dorsi of them (Figure 4B). We further performed western blot analysis of the *Mb* gene at the protein expression level, and grey scale analysis of the assay results showed that the protein expression was higher in Bama pigs than Yorkshire pigs (Figure 4C).

### 3.5. qRT-PCR Validation of Transcription Factors on Porcine Mb Gene Regulatory Network of the Bama Breed

**Figure 5:**
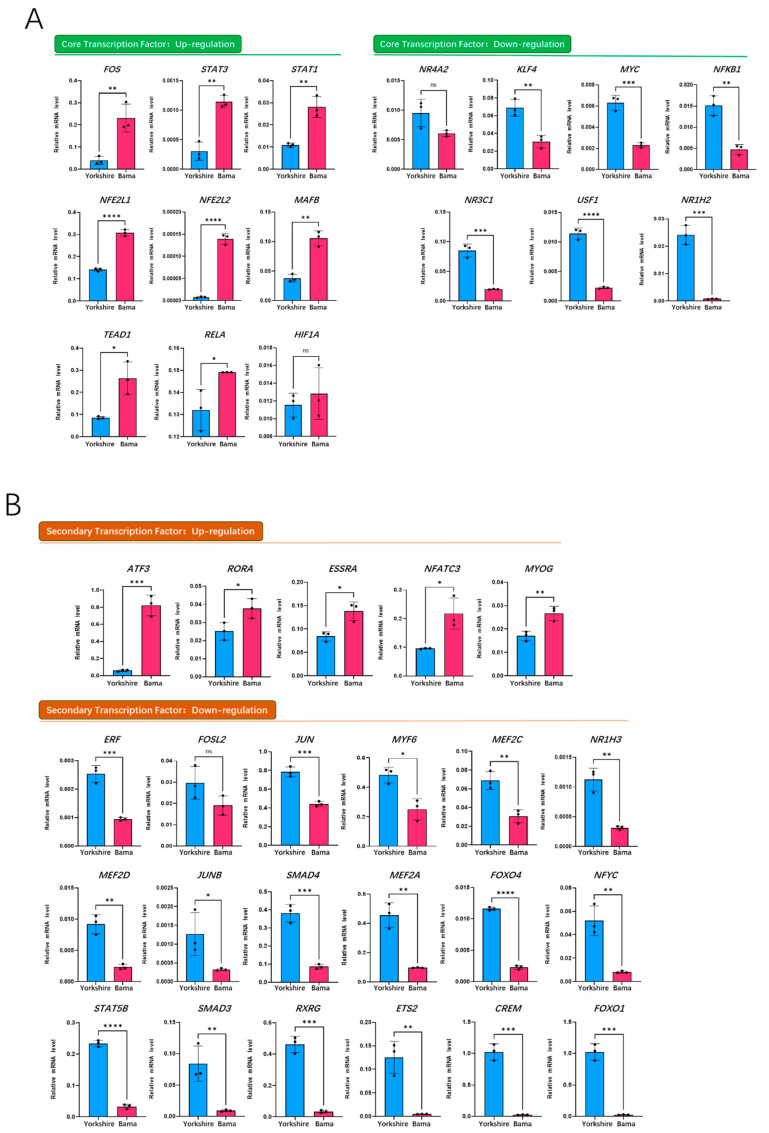

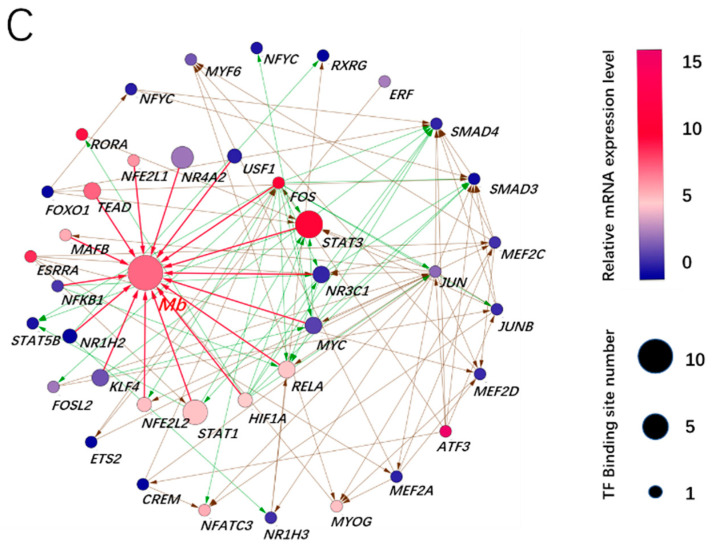
qRT-PCR validation of transcription factors on the porcine *Mb* gene regulatory network of the Bama breed. (**A**) core transcription factor expression of the Bama breed in the Mb-TFs network; (**B**) secondary transcription factor expression of the Bama breed in the Mb-TFs network; (**C**) overview of transcription factor expression foldchange and binding sites number of the Bama breed in the Mb-TFs network. * *p* < 0.05, ** *p* < 0.01, *** *p* < 0.001, **** *p* < 0.0001, ns: no significance.

To further identify the specifically up-regulated core and secondary transcription factors of *Mb* gene in the Bama breed, we verified the mRNA expression of all transcription factors on the PPI network by qRT-PCR. The results showed that among the eight up-regulated TFs, the mRNA expression of *FOS*, *STAT3*, *TEAD1* and *NFE2L1* increased more than two-fold (Figure 5A). Among the secondary TFs, the mRNA expression of most TFs was down-regulated (Figure 5B), and only the mRNA expression levels of *ATF3*, *RORA* and *ESRRA* were up-regulated by more than two-fold (Figure 5B).

Based on Figure 5A, we deduced that *STAT3* was the most important core transcription factor, due to its seven-fold increase in expression and seven binding sites, whereas *ATF3* was the most important initiator of *Mb* gene expression, due to its 13-fold increase in expression and as an upstream regulator of *STAT3*.

## 4. Discussion

*Mb* is the key protein responsible for meat color formation, although other heme protein such as hemoglobin and cytochrome C may also play a role in the color of beef, mutton, pork and poultry. The in-depth explanations on the regulatory mechanism of the *Mb* gene in pigs has been limited by the lack of genomic information. Our research is mainly trying to predict the activation of *Mb* gene from transcription factors by mining genome and transcriptome data in public databases.

The research on meat quality has always been the most important field in pig production. Meat purchasing decisions are influenced by color more than any other quality factor, as consumers use discoloration as an indicator of freshness and health. As a result, nearly 15% of retail beef is discounted in price due to surface discoloration, which equates to annual revenue losses of $1 billion [7,28]. The economic improvement related to meat products and maintaining the color of meat products depends on our understanding of the pre- and post-mortem myoglobin. 

In recent years, *Mb* has received more attention mainly in the field of meat product production, because it is the sarcoplasmic haemoglobin protein primarily responsible for the color of meat obtained from bleeding livestock carcasses [29]. The resonant nature of the conjugated double bond in the haemoglobin group is responsible for *Mb*’s ability to absorb visible light, and thus perform its function as a pigment [30,31]. The chemistry and function of *Mb* in live muscle and meat products may be different. In live muscle, *Mb* acts as an oxygen-binding agent, delivering oxygen to the mitochondria and allowing the tissue to maintain its physiological function [32,33,34]. In meat products, *Mb* serves as the primary pigment responsible for the red meat color. Although the bleeding of food animals at harvest removes the primary color of blood, some residual blood gets trapped inside the arteries and veins within the large skeletal muscles, resulting in the presence of haemoglobin in meat.

The peroxides produced by lipid oxidation in muscle foods can not only lead to the deterioration of food nutritional texture and flavor sensory quality, but also produce carcinogens [35]. At present, it is agreed in the field of food research that hemoglobin (hemoglobin and myoglobin), especially myoglobin, is considered to be the most important endogenous lipid oxidation factor of lipid peroxidation. For example, our experimental results show that the content of *Mb* in the muscle of local pig breeds is higher than that of European commercial pig breeds, but in fact, local pig breeds do not have meat fission because of the high expression of *Mb*. We speculate that there are some anti-lipid oxidation factors in the muscle of local pig breeds. Some transcription factors on the Mb-TFs network deserve attention.

Although *FOS* is the most up-regulated gene in Bama pigs’ Mb-TFs network, in fact it has only one binding site on the promoter of the *Mb* gene. As a nuclear protein transcription factor, *FOS* plays an important role in regulating cell growth, division, proliferation, differentiation and even programmed death. *FOS* also plays an important role in myogenic differentiation [36]. *FOS* inhibits the expression of *MyoD* in myoblast division by binding to the hypothetical cAMP (cyclic adenosine monophosphate) response element on the *MyoD* promoter, thus inhibiting myoblast in mice [37]. Ten trans eQTL (expression quantitative trait locus) genomic regions were found in one study. Among them, potential genes regulating lipid metabolism were found in three regions [38].

Studies in the past years have shown that many cytokines and hormones regulate lipid metabolism by activating JAK-STAT signaling pathways. Activated STATs can directly regulate lipid metabolism by affecting the expression of enzymes. *STAT1* and *STAT3* are not only significantly up-regulated, but also have more binding sites (*STAT1:6*, *STAT3:7*) at the promoter region of *Mb* in our results. *STAT1* and *STAT3* are signal transducers and transcriptional activators that play an important role in regulating cell proliferation, differentiation and migration [39,40]. Grohmann and others have shown that oxidative stress caused by obesity inhibits the activity of protein tyrosine phosphatase and leads to the activation of *STAT1* [41]. Meng et al. compared the meat quality of three cattle breeds. The results showed that the activation of *STAT3* was beneficial to improving the meat quality of cattle. Guillet-Deniau et al. suggested that activation of the JAK-STAT pathway may be associated with myogenic differentiation. Skeletal muscle serotonin increases myostatin expression and triggers activation of the JAK-STAT pathway in primary myogenic cells [42]. Interestingly, the gene with the largest up-regulation multiple on the Mb-TFs network is *ATF3*, which belongs to the secondary transcription factor of *Mb* and does not bind to the promoter of *Mb*. Glal et al. found that *IL-22* can up-regulate *ATF3* in intestinal epithelial cells, and *ATF3* is necessary for transmitting *IL-22* signal to lead to *STAT3* phosphorylation and subsequent AMP (Adenosine monophosphate) induction. *ATF3* itself does not directly act on *STAT3*. On the contrary, *ATF3* regulates *p*-*STAT3*, including *SHP2* and *PTP-Meg2*, by negatively targeting protein tyrosine phosphatase (PTPs) [43]. 

In addition, although *NFE2L2* and *NFE2L1* do not have many up-regulated multiple and binding sites, as *CNC-bZIP* family transcription factors with basic lysine zipper structures, they play an important role in resisting oxidative stress and maintaining redox homeostasis. Hou et al. found that subcutaneous adipose tissue (SAT) of a series of adipocyte-specific *NFE2L1* knockout (*NFE2L1* (f)-KO) mice significantly reduced mass insulin resistance adipocyte hypertrophy and severe adipose inflammation. The results of *NFE2L1* deficiency may interfere with the expression of lipolysis genes in adipocytes, lead to adipocyte hypertrophy, and then lead to inflammation, cell pyrogenesis and insulin resistance [44]. *NFE2L2* deficiency in hepatocytes inhibits the expression of peroxisome proliferator-activated receptor gamma (*PPAR γ)* and its downstream adipogenic genes in the liver and/or primary hepatocytes induced by HFD (High Fat Diet) and palmitate exposure. However, gene activation through *NFE2L2* can be blocked by small MAF proteins, such as MAFG and MAFK, to balance *NFE2L2* action and regulate the intracellular oxidation levels [45]. In our study, we found that *MAFB* was up-regulated simultaneously with *Mb* and *NFE2L2* in the longissimus dorsi of local pigs. *MAFB* is a transcription factor that plays a regulatory role in muscle development and lipid metabolism. It is a member of the MAF transcription factor family. Its members contain a basic leucine zipper (bZIP) domain that binds to DNA. These bZIP domains are located in the MAF recognition element (MARE) and regulate the transcription of the target gene by binding to the acidic domain in the gene [46]. Recently, Hamada et al. have shown that *MAFB* expression is induced by the liver X receptor (*LXR*), which is activated by oxidized low-density lipoprotein (LDL) and regulates the macrophage apoptosis inhibitor (AIM), and adipose tissue macrophages (ATM). The expression of AIM in adipocytes plays a role in lipolysis of adipocytes [47]. Tran et al. reconstructed the hematopoietic system of mice by transplanting *MAFB* -/- fetal liver cells, and feeding these mice with a high-fat diet can inhibit the increase in fat mass [48]. In addition, Mahmassani et al. demonstrated that unique up-regulated genes expressed in old muscle after bed rest indicated increased inflammation and muscle wasting (*CXCL2*, *GADD45A*) and decreased *MAFB* correlated with the change in leg lean mass [49]. The promoter region of *Mb* gene contains only one *MAFB* binding site, so its regulatory effect deserves further attention.

As high-throughput public data published on the NCBI (National Center for Biotechnology Information) increases, a large number of pig transcriptome data are expected to contribute to the construction of the Mb-TFs network. At present, local pig breeds in China are distributed in six major regions (South Chinese, Central China, North China, Lower Yangtze River Basin, Southwest, and Plateau) [50]. Unfortunately, we selected expression profile datasets from only two local Chinese pig breeds (Dapulian: North China and Wei: Lower Yangtze River Basin) to construct the Mb-TFs network, which resulted in a less precise number of core transcription factors for the screened Mb-TFs network. In our view, the introduction of at least six geographically representative pig breeds into the dynamic Mb-TFs network will greatly improve the credibility of the gene interaction, although many institutions have long been engaged in in vitro and in vivo experiments comparing the meat quality of local pig breeds and commercial pig breeds, as well as large-scale genomic and transcriptomic investigations.

In our preliminary experiments, we also examined the transcriptome data of some local pig breeds in public databases, and principal component analysis revealed that only a few samples in the dataset could meet the threshold for differential gene expression analysis. In addition, another reason for the scarcity of studies on pig meat color is that the *Mb* gene is not found in the pig genome reference annotation file (*GFF*), and genome aligners such as *HISAT2* and *BWA* are unable to perform *SNP* calling and calculate reads for the *Mb* gene. The above problems can only be solved by relying on the database to accumulate more and more high-throughput sequencing data of local pig muscle tissues in the future.

## 5. Conclusions

To summarize, through data mining, we found the complete genomic information of the *Mb* gene, constructed the transcription model of the *Mb* gene and identified the potential specific activation transcription factors in the Bama pig breed through in vivo experiments. 

Our research strategy provides an experimental basis for further research, revealing the formation of pig meat color and its relationship with molecular mechanisms on lipid metabolism.

## Data Availability

Data are available upon a reasonable request.

## Supplementary Materials

The following are available online at https://www.mdpi.com/article/10.3390/ani11123599/s1. Table S1: Gene Primer Sequences; Figure S1: Original Western Blot Figures.
