# Supplementary figures and images for: Prediction and Experimental Verification of a Hierarchical Transcription Factor Regulatory Network of Porcine Myoglobin (Mb)
Source: Animals (Basel). 2021 Dec 19;11(12):3599. doi: 10.3390/ani11123599 (PMC8698129; doi:10.3390/ani11123599)

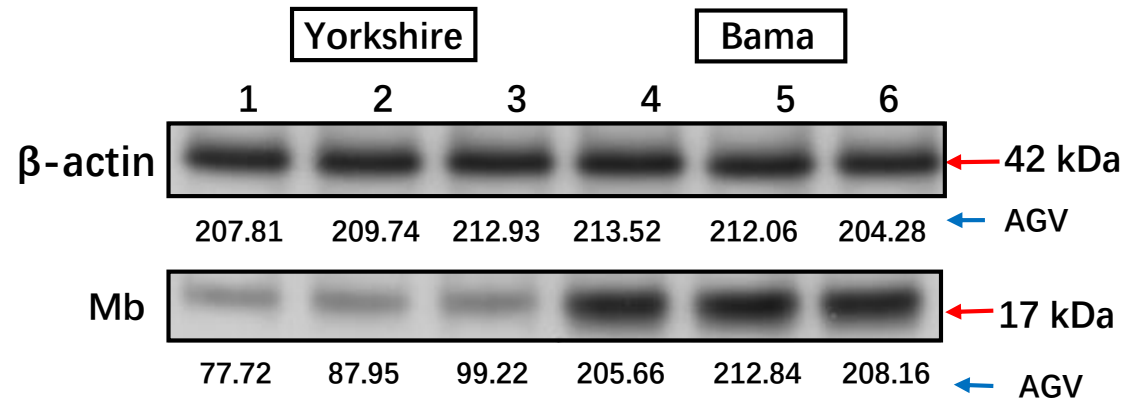

Average Gray Value : AGV

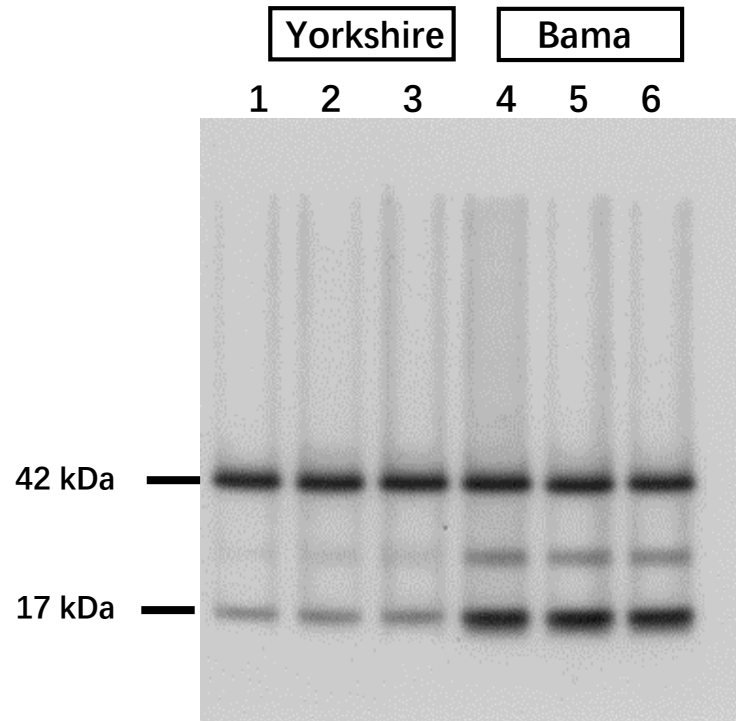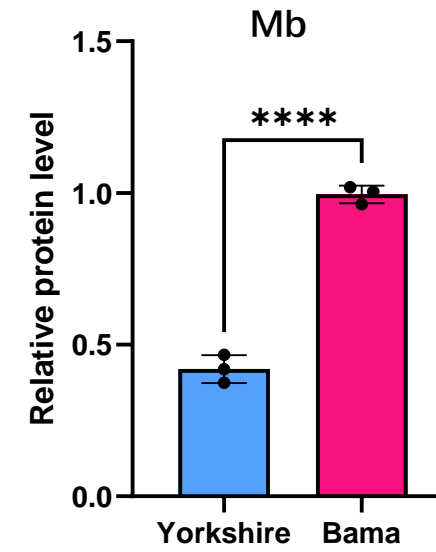

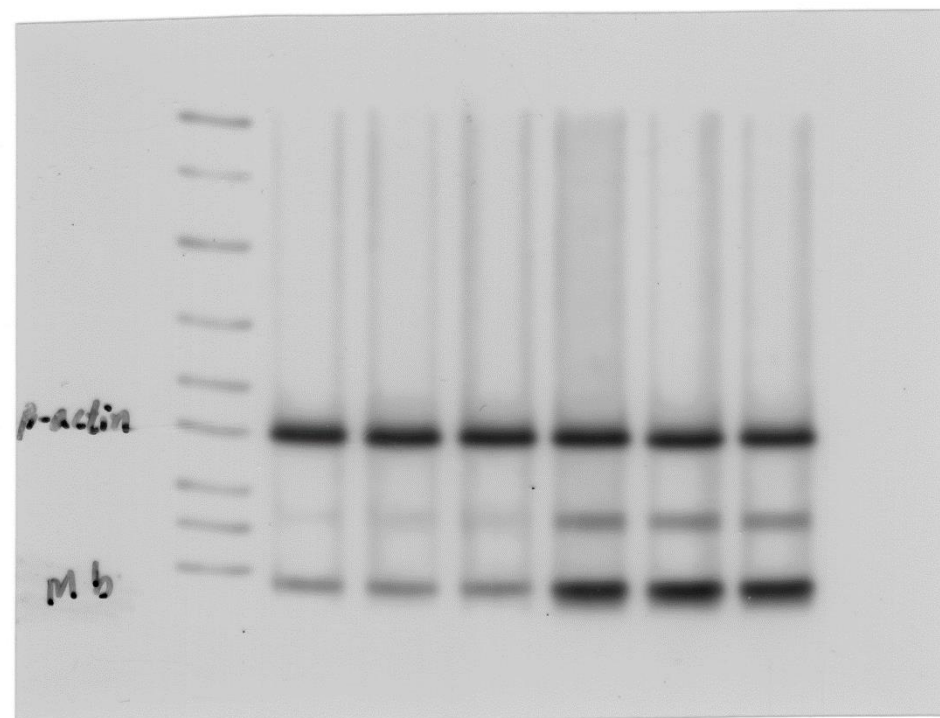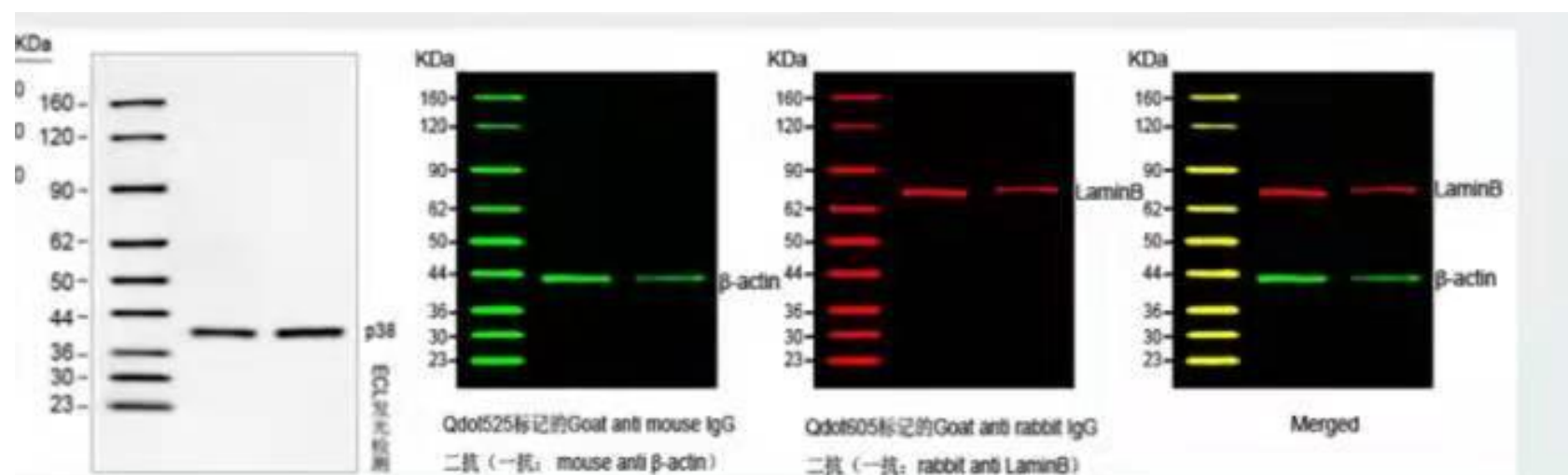

Supplement: Supplementary file 1 [file animals-11-03599-s001.zip › animals-1470452-supplementary/Western Blot-12.15.pdf]
